# Relationship between Nutritional Status and Gastrointestinal Symptoms in Geriatric Patients with End-Stage Renal Disease on Dialysis

**DOI:** 10.3390/nu10040425

**Published:** 2018-03-29

**Authors:** Dinorah Carrera-Jiménez, Paola Miranda-Alatriste, Ximena Atilano-Carsi, Ricardo Correa-Rotter, Ángeles Espinosa-Cuevas

**Affiliations:** 1 Nephrology and Mineral Metabolism Department, Instituto Nacional de Ciencias Médicas y Nutrición Salvador Zubirán, Mexico City 14080, Mexico; nut.dcarrera@gmail.com (D.C.-J.); pvma2000@hotmail.com (P.M.-A.); xime1879@hotmail.com (X.A.-C.); correarotter@gmail.com (R.C.-R.); 2 Health Care Department, Autonomous Metropolitan University, Mexico City 04960, Mexico

**Keywords:** nutritional status, gastrointestinal symptoms, geriatric patients, dialytic therapy.

## Abstract

Gastrointestinal symptoms (GIS) are common in patients with end-stage renal disease (ESRD) and are associated with nutritional risks resulting from low food intake. Little is known about the relationship between GIS and malnutrition in geriatric patients with ESRD. The main objective of this study was to determine the relationship between nutritional status and severity of GIS in geriatric patients on dialysis therapy. Clinically-stable geriatric patients (older than 60 years old) who were dialysis outpatients were included in this cross-sectional study. The severity of GIS was assessed using the Gastrointestinal Symptoms Questionnaire (GSQ, short version), with patients classified into three groups: mild, moderate, and severe. Nutritional status was evaluated with the Malnutrition Inflammation Score (MIS), anthropometric assessment, biochemical parameters, and bioelectrical impedance. Descriptive statistics were used and differences between groups were analyzed with ANOVA and Kruskal Wallis, with a *p* < 0.05 considered to indicate significance. Fifty patients completed the study; the median age was 67 years old. Twenty-three patients were on hemodialysis (HD) and 27 were on peritoneal dialysis (PD). No significant differences were found according to dialysis modality, presence of diabetes, or gender. Ninety percent of patients had at least one GIS. Poorer nutritional status (evaluated by MIS) was related to a higher severity of GIS. There were no significant differences with other nutritional parameters. Our study showed a high prevalence of GIS in geriatric patients. There were no differences in observed GIS values that were attributed to dialysis modality, gender, or presence of type 2 diabetes mellitus (DM2). Severe GIS values were associated with poorer nutritional status determined by MIS, however, there was no association with anthropometry, biochemical values, or bioimpedance vector analysis.

## 1. Introduction

Gastrointestinal symptoms (GIS) are common among patients with end-stage renal disease (ESRD), in particular in those undergoing renal replacement therapy and with a prevalence of 32–85% [1,2,3,4,5,6,7,8,9,10,11,12,13]. In México it is estimated that 28% of ESRD patients on dialysis are older than 60 years old [14]. GIS can significantly affect the ingestion, digestion, and absorption of nutrients, thus resulting in the deterioration of nutritional status [1,5,15]. These symptoms may be attributed to uremia, some comorbidities (in particular diabetes mellitus), the effect of dialysis, diverse pharmacological treatments, and changes in diet and lifestyle [1,3,16,17]. 

GIS are common in older adults and are related to bowel disorders caused, on one hand, by the organic and structural changes associated with aging and, on the other hand, by the effect of pathological conditions such as diabetes, pancreatic or liver disease, and polypharmacy. The changes described in the basic digestive functions during aging are delayed motility, a decrease in gastric secretions, alteration of the mucosal-bicarbonate barrier, an abnormal intraluminal digestion, and a decreased absorption [18,19]. These changes represent underlying mechanisms responsible for symptomatic gastrointestinal dysfunctions such as dysphagia, gastroesophageal reflux disease, dyspepsia, irritable bowel syndrome, constipation, indigestion, and decreased nutrient absorption, all of which affect the nutritional status of older adults and expose them to the risk of malnutrition [19,20].

Malnutrition is commonly reported in renal patients, most frequently in older adults, and is attributed to a global energy-protein deficiency [21]. The insufficient intake of nutrients and energy can be related to the presence of gastrointestinal disorders and altered appetite [22,23]. Approximately 12.5% of patients with chronic diseases like diabetes mellitus, cancer, and cardiac disorders, among others, have a lower weight than what is normally considered to be adequate and therefore present higher nutritional risk [23].

In addition to the above mentioned complications, a renal patient´s nutritional status is affected by the presence of protein energy wasting (PEW), which is caused by the pathophysiological relationship between malnutrition, inflammation, and atherosclerosis. This condition is linked to cardiovascular diseases, which collectively are the cause of the highest number of deaths among these patients [24]. PEW is characterized by a set of mechanisms and conditions that include malnutrition, inflammation, metabolic acidosis, accelerated catabolism, endocrine alterations (abnormal metabolism of amino acids, insulin, growth hormone, and some minerals), symptoms related to uremic syndrome, loss of nutrients in the dialysate, as well as the complications of concomitant diseases. PEW may cause infection, cardiovascular disease, frailty, and depression, but these complications may also increase the extent of PEW. A consequence of inflammation is decreased appetite due to elevated cytokines, and it is likely to also impact the presence of some gastrointestinal symptoms [24,25,26].

Together with frailty and decreased functional capacity, older adults are at higher risk of complications such as anemia and protein energy wasting (PEW) [27,28,29]. Although GIS have been reported to be a possible cause of the nutritional status deterioration in ESRD patients, few studies have directly evaluated their relationship and their impact on elderly patients [4,10]. The aim of this study was to determine the relationship between the nutritional status and presence and severity of GIS in elderly patients on dialysis therapy.

## 2. Materials and Methods 

This was a cross-sectional study in a clinically stable group of geriatric patients that was performed from April 2015 to May 2016. The study was approved by the ethics committee “Institutional Committee for Biomedical Research in Humans”, on 31 March 2015 (NMM-1462-15/16-1). Patients undergoing hemodialysis (HD) or peritoneal dialysis (PD) at our institution were invited to participate either by phone or in person at the hospital and those who accepted where given an appointment for the first assessment. Informed consent was obtained from all patients.

We included ESRD patients older than 60 years old who had been receiving dialysis for at least 3 months. Patients diagnosed with a gastrointestinal disease, infectious illness, who were hospitalized in the last month, fed by catheter, or were unwilling to participate in the study were excluded. Assessment of GIS and nutritional status was performed by a trained nutritionist.

### 2.1. Gastrointestinal Symptoms Assessment

To evaluate the presence and severity of GIS we used a modified Gastrointestinal Symptoms Questionnaire (GSQ) in its short version. This is an eight-item questionnaire that takes into account the presence of common GIS in ESRD (abdominal pain, bloating, pyrosis, nausea, vomiting, constipation, and diarrhea) and loss of appetite or anorexia during the last 4 weeks. The severity of symptoms was defined according to their intensity and impact on daily activities with a five-grade Likert scale, where 1 means “no discomfort” and 5 indicates “unbearable discomfort”. The higher the overall score, the more pronounced the symptoms. Patients were classified into three groups: Group 1, mild symptoms (9–10 points); Group 2, moderate symptoms (11–13 points); and Group 3, severe symptoms (≥14 points) [4,30]. The presence of GIS was defined as the patient reporting at least one symptom.

### 2.2. Nutritional Status

Nutritional status was evaluated with the Malnutrition Inflammation Score (MIS) tool, which has 10 components divided into four sections: nutritional history (weight change, dietary intake, GIS, comorbidity according to time on dialysis, functional capacity), physical examination (subcutaneous fat and muscle wasting), body mass index (BMI), and biochemical parameters (serum albumin level, and iron binding capacity or serum transferrin level). Each item has four levels of severity (0 = normal, 1 = mild, 2 = moderate, 3 = severe). The sum of all items ranges from 0 = normal to 30 = severely malnourished; a higher score reflects a more severe degree of malnutrition and inflammation [31]. We also assessed the presence of PEW by the International Society of Renal Nutrition and Metabolism (ISRNM) diagnostic criteria, which requires that patients meet at least one criterion in three of the four proposed categories: altered biochemical criteria, loss of body mass, loss of muscle mass, and decreased protein or energy intake [24].

Body composition and hydration status were evaluated using the bioelectrical impedance vector analysis (BIVA) on the day of the peritoneal equilibrium test in PD patients and at the end of the hemodialysis session for HD patients. Bioimpedance measurements were obtained with a Bodystat QuadScan 4000 equipment (Bodystat limited, Isle of Man, UK), which employs the standard tetrapolar technique (two pairs of skin electrodes on hand and foot) at a frequency of 50 kHz. Vector position on the resistance (R) and reactance (Xc), RXc graph is interpreted as follows: vector displacements parallel to the major axis of tolerance ellipses, out of the normal poles of 50% and 75%, indicate progressive changes in tissue hydration (dehydration out of the upper poles, and over-hydration with apparent edema, out of the lower poles); vectors lying on the left or right side of the major axis indicate more or less cell mass, respectively [32,33]. 

In addition, other nutritional markers were measured and analyzed: anthropometry (mid-arm circumference and triceps skinfold thickness, to estimate arm muscle circumference), biochemical parameters (serum creatinine, blood urea nitrogen, hemoglobin, serum albumin). Handgrip strength was used to estimate the muscle function with a mechanical Smedley III dynamometer (Takei, Niigata, Japan) [34]. Three measurements were made on each hand; the greatest value was taken into account. Patients were asked to register a 24-h recall to assess energy and nutrient intake. Protein and energy intake was calculated using the nutrient software program NutriKcal^®^ (Cosinfo SC, CDMX, Mexico).

### 2.3. Statistical Analysis

Continuous variables were expressed as mean ± standard deviation and categorical variables were presented as absolute (number of participants) and relative frequencies (percentages). Statistical analysis was performed with SPSS, version 19.0 (IBM, San Jose, CA, USA). Student’s *t*-tests and ANOVA were used to compare continuous variables between groups; X^2^ and Kruskal Wallis were employed for the comparison of non-parametric variables and Pearson’s X^2^ or Fisher’s exact were used for categorical variables. A significant difference was considered when *p* < 0.05.

## 3. Results

**Figure 1:**
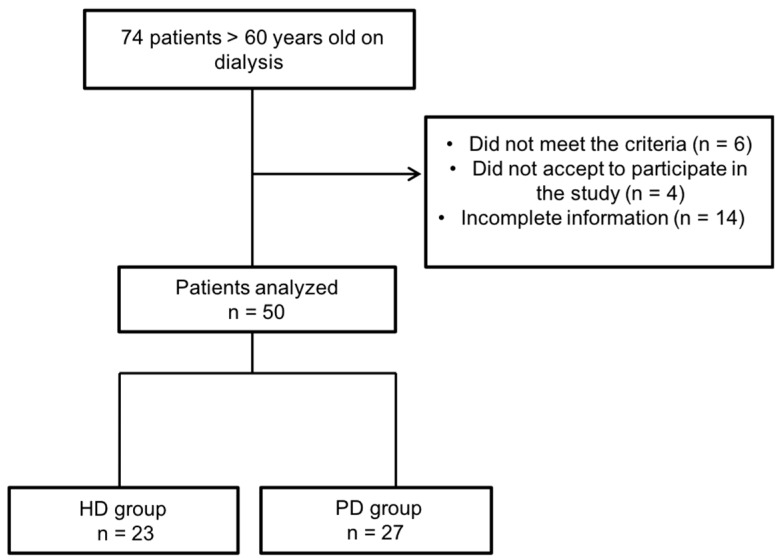
Flowchart of the study selection process. HD, hemodialysis; PD, peritoneal dialysis.

Of 74 patients invited to participate, 10 patients were excluded, as they did not meet the inclusion or had exclusion criteria, and 14 were eliminated during the study for various reasons, leaving a total of 50 patients for the analysis (Figure 1).

Characteristics of the study population are shown in Table 1. The prevalence of type 2 diabetes mellitus (DM2) corresponds to 66% of the total patients: 70% in HD and 63% in PD. Of the total population, 90% reported the presence of at least one GIS. The median age of participants was 67 years. When dividing the population by age range (60 to 65 years, 66 to 74 years, and ≥75 years), no significant differences were found in the prevalence or severity of the GIS (with GIS scores of 13.5, 12.1, and 12.1, respectively (*p* = 0.35)). Additionally, there was a difference in GIS severity only in women (*p* < 0.02) but not in men (*p* > 0.06), and there were no differences according to gender in regard to total GIS score between women and men (*p* = 0.8), or presence of diabetes (*p* = 0.1). Regarding time on dialysis, the population was divided into three groups according to time on dialysis: <1 year, from 1 to 4 years, and >4 years; we did not find differences in the score of severity of GIS according to time on dialysis (*p* = 0.9). Forty percent of the patients reported the use of some drug(s) for the relief of their GIS: 19 patients (38%) used antacids, 5 patients (10%) used sennosides, only 1 patient (2%) reported frequent use of probiotics; only 8 patients (16%) reported consistent use of phosphate binders. 

Even though there appear to be differences in severity of GIS according to number of months of dialysis, continuous data with a Spearmen's correlation show no association between this and the variables (correlation coefficient 0.016, *p* = 0.9).

### 3.1. Gastrointestinal Symptoms

**Figure 2:**
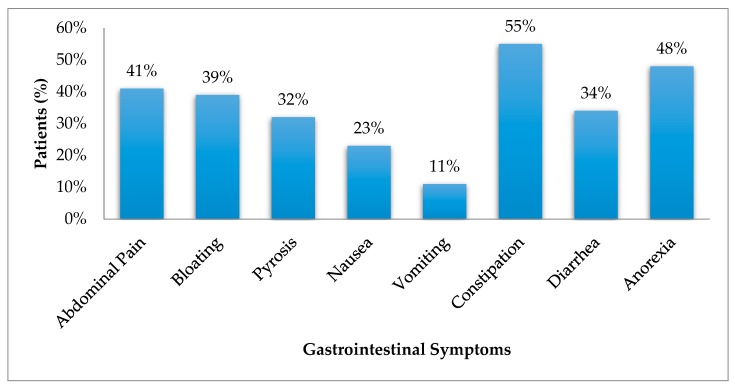
Prevalence of GIS in patients older than 60 years on dialysis.

The prevalence of constipation, anorexia, and abdominal pain was higher than other GIS, while vomiting was the least prevalent. (Figure 2)

Of the total population, 11 patients (24%) had mild symptoms, 14 patients (31%) had moderate symptoms, and 20 patients (44%) had severe symptoms (Table 2) The mean GIS severity score was 12.9 ± 3.5 with a range of 8 to 23 points throughout the population; for each group, the mean score was: 9 ± 0.8 for Group 1; 12.1 ± 0.8 for Group 2; and 16.4 ± 2.7 for Group 3. 

The prevalence of constipation and nausea was significantly higher in the group with severe symptoms compared with that of mild ones; while abdominal pain was significantly greater in the severe group compared with the groups of mild and moderate symptoms. The prevalence of bloating, heartburn, vomiting, diarrhea, and anorexia was not different between the groups.

### 3.2. Nutritional Status

**Figure 3:**
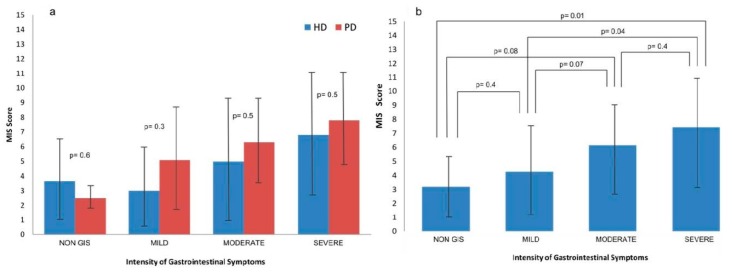
Prevalence of malnutrition (MIS) and the severity of GIS according to (**a**) dialysis modality, (**b**) all patients in renal replacement therapy together.

Regarding the nutritional status evaluated by the Malnutrition Inflammation Score (MIS), the higher the severity of GIS, the higher the degree of malnourishment. When comparing the severity of GIS by dialysis modality, we did not find significant differences between patients undergoing hemodialysis versus patients on peritoneal dialysis (Figure 3a). The MIS score was significantly higher in patients with severe symptoms (Group 3) as compared to the patients with no GIS or mild GIS (Group 1), but there were not differences compared to the group with moderate symptoms (Group 2). No differences were found between the groups of patients without symptoms and those with mild symptoms, but a tendency (*p* = 0.07) was observed when comparing them with those with moderate symptoms (Figure 3b). 

We compared nutritional parameters, such as anthropometric and biochemical markers, and found that there were no significant differences between groups. Wasting was present in 22% of patients, according to the criteria used to diagnose PEW, and serum chemistry and muscle mass were altered in all of the patients; with respect to body mass, only four patients had a BMI ≥ 23, six patients consumed less energy than recommended. In a binary logistic regression analysis, no association was found between GIS and wasting (r = 0.4, *p* = 0.5) (data not shown). 

Patients completed a 24-h record of their global energy intake, with the results indicating that the patients averaged 1441 kcal per day (22 kcal/kg), while average protein intake was 0.8 g/kg; patients with moderate symptoms had significantly lower protein intake (grams per kg) than the other groups (Table 3). We also compared energy and nutrients intake between patients on hemodialysis and patients on peritoneal dialysis, and we observed that energy intake was significantly lower in the group on peritoneal dialysis than in hemodialysis patients (1238 ± 352 vs. 1684 ± 579 kcal/day, *p* = 0.003 and 19.3 ± 7.4 vs. 25.2 ± 9.5 kcal/kg, *p* = 0.02) (data not shown). No differences were observed in energy, lipids, or carbohydrate intake between the groups. According to the bioelectrical impedance evaluation and vector phase angle there were not significant differences between groups (Table 3).

#### Impedance Vectors 

**Figure 4:**
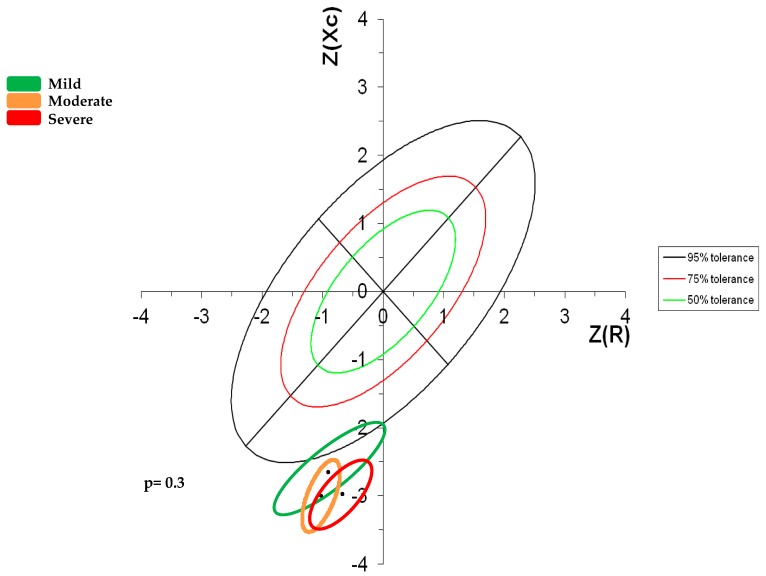
Z Score of bioelectric impedance vectors in geriatric patients according to the severity of GIS.

Figure 4 shows the bivariate distribution of the standard deviates of resistance/height (R/H) and reactance/height (Xc/H), both with zero mean, unit standard deviation, and correlation coefficient from a healthy population according to the RXc score graph methodology [33]. The two standard deviates of the bivariate Z-score vector are calculated from individual R/H and Xc/H data as Z(R) = (R/H − the mean value)/SD, and Z(Xc) = (Xc/H − the mean value)/SD, that is with respect to the means and standard deviations of a specific, reference population by race, gender, and age range. The long axis of the Z score ellipse represents hydration status, whereas the short axis represents body tissue; the upper side of the long axis represents deficit of hydration status (dehydration) and the low side of the long axis represents excess of hydration status (anasarca), the right side of the short axis represents loss of tissue (muscle at the top right side and fat at the low right side), the left side of the short axis represents excess of tissue (excess of muscle at the top side and excess of fat at the low left side). This RXC score graph methodology represents simultaneously tissue and hydration status of a patient with volume overload as our patients. In our Z score graph it was observed that the three groups that had been assigned according to severity of GIS are in the lower right quadrant, out of the 75% pole, indicating that loss of tissue (fat) and volume overload occurred regardless of the severity of the GIS. 

## 4. Discussion

The high prevalence of symptoms observed in our population is consistent with that found in other studies [1,3,12,13]. Most studies take into account patients of all ages, so that few consider being older as a risk factor in the presence and severity of GIS [7,27]. However, in this study, the increase in the age range did not influence either the higher prevalence or severity of symptoms.

Diabetes mellitus type 2 (DM2) is one of the main causes of chronic kidney disease and the relationship between the alterations caused by this disease and the presence and severity of GIS has been described before [4,35]. Some studies did not observe differences in the frequency of symptoms among patients with and without DM2 [3,36]. In our study, there were no differences in the prevalence and severity of GIS in patients with DM2 compared with those without it.

Normalized energy and protein consumption per kilogram of ideal weight was assessed to see if they complied with the established recommendations by the international Kidney Disease: Improving Global Outcomes (KDIGO) guidelines. We found that neither protein nor energy intake cover the requirements recommended for patients on dialysis, which therefore puts them at a higher risk of malnutrition [37,38]. Patients on PD had lower energy intake than HD patients. When comparing nutrient and energy intake by severity of the symptoms, the group with moderate symptoms had lower protein (g/kg) intake than the groups with mild and severe symptoms; we think this might be due to the higher proportion of patients on PD in the moderate GIS group. Patients on PD attend the clinic less frequently than those on HD, so they are less likely to follow the recommendations of a nutritionist; on the other hand, patients on HD visit the dialysis clinic at least twice a week, therefore they are better monitored.

In this study, the prevalence of symptoms was independent of the type of dialysis. There are inconsistencies in the literature comparing the prevalence of GIS in patients with CKD by type of dialysis [1,3,6,7,8,11]. These contradictions may be due to the use of different questionnaires and to the type of symptoms that they take into account since, depending on the type of dialysis, they may affect different segments of the digestive tract. In this study, the questionnaire we used takes into account symptoms present in both segments of the digestive tract [39]. Anorexia is a common symptom in dialysis patients and is associated with poor nutritional status and the risk of presenting comorbidities [40,41]. Even though anorexia was one of the most prevalent symptoms in our population, we did not find an association between the severity of GIS and loss of appetite. 

There are few studies that relate the severity of GIS to the degree of malnutrition [1,4]. Li et al. correlated the severity of GIS with nutritional status according to the Subjective Global Assessment (SGA) and other anthropometric and biochemical indicators, finding greater malnutrition in the group with severe symptoms when using SGA but not with the other nutritional parameters [4]. In our study, using the MIS tool, we found a higher prevalence of malnutrition in the group of patients with severe symptoms than in the mild group, however, we found no differences with wasting and other nutritional parameters. Although the MIS tool includes within its variables the presence of GIS and this could generate an error of interpretation of the results of our study, we also used a tool (GSQ) that allowed us to evaluate the severity of these symptoms. The analysis of the results of the present study was made based on the severity of the symptoms rather than the presence of them. 

In the literature, it is accepted that BIVA has the potential to evaluate hydration and nutritional status in several clinical conditions like liver diseases in critical care patients, renal diseases, hemodialysis, peritoneal dialysis, and cardiac failure [32,33]. In this study, we evaluated the distribution of impedance vectors between the three groups according to GSQ classification; we observed that regardless of the severity of the symptoms our patients were located in the lower right quadrant, outside the recommended range, which represents the presence of cachexia and over-hydration. Our results show that the nutritional status of patients with mild GIS is not necessarily better than that of the patients with severe symptoms. In fact, regardless of the severity of the symptoms, the three groups appear to present nutritional risk; there are no significant differences between them. We believe it is possible that discrepancies observed between the nutritional parameters and the severity of GIS are due to the age of the studied patients and to comorbidities of CKD that put them at nutritional risk, independent to the severity of their symptoms, and are also due to a bias in the self-report of the severity of the symptoms caused by the subjectivity in the perception of malaise, which made it difficult to accurately evaluate the symptomatology and therefore determine the classification of groups. 

We believe that the self-assessment of the severity of our patients' symptoms was influenced by the chronic maintenance of the intensity of the symptoms, that is, they became accustomed to feeling their symptoms so they cannot distinguish their severity unless a symptom is aggravated [42]. Age could be another factor of bias in the evaluation of symptoms, since although the questionnaire was carefully explained to each patient, some showed difficulty in evaluating the severity of their symptoms. Different studies agree that older adults adopt a more conservative attitude towards the sensation of pain and are more reluctant to report it when it occurs [43].

One of the strengths of our study is that this is one of the few studies comparing the prevalence of GIS specifically in the geriatric population on dialysis. A limitation of this study is that, in comparison with other studies, the number of patients was low and difficulties were encountered during the uptake of the patients. Another limitation was that we did not use a three-day dietary record to estimate energy and nutrient intake because patients did not complete it; this prevented us from performing other analyses.

## 5. Conclusions

Geriatric patients in dialysis have a high prevalence of GIS (90%). There were no significant differences between the presence of symptoms and the type of dialysis. Patients with severe symptoms presented with increased constipation, abdominal pain, and nausea. A positive association was found between the MIS tool score and the severity of the symptoms; however, with other nutritional indicators such as anthropometric and biochemical data, it was not possible to prove that nutritional status was affected by the severity of GIS. Studies with more patients and a prospective study are required to confirm that nutritional status is influenced by the severity of GIS in geriatric patients with dialytic therapy.

## Figures and Tables

**Table 1:** General characteristics of the study population by severity of GIS.

Variable	Total *n* = 50	No GIS *n* = 5	Mild *n* = 11	Moderate *n* = 14	Severe *n* = 20	*p*
^₫^ Women, *n* (%)	22 (44) *	1 (20)	8 (73)	3 (21)	10 (50)	0.02
^₫^ Men, *n* (%)	28 (56)	4 (80)	3 (27)	11 (79)	10 (50)	0.06
^µ^ Age (years)	67 (60–84)	65 (62–83)	69 (60–78)	65 (60–82)	68 (60–84)	0.4
^µ^ Time on Dialysis (months)	20 (3–96)	13 (3–42)	14 (3–48)	20.5 (5–96)	31.5 (3–84)	0.4
^₫^ DM2, *n* (%)	33 (66)	5 (100)	5 (45)	11 (79)	13 (65)	0.1
^₫^ Use of drugs for GIS, *n* (%)	20 (40)	1(20)	4 (36)	6 (43)	8 (40)	0.1

DM2, type 2 diabetes mellitus; body mass index (BMI); GIS, gastrointestinal symptoms. * Differences in GIS score between men and women. ^₫^ Data is expressed in number of patients (percentages). ^µ^ Data is expressed in medians (interquartile range).

**Table 2:** Prevalence and severity of Gastrointestinal Symptoms.

Gastrointestinal Symptoms	Intensity of the Symptoms			
	Mild	Moderate	Severe	*p*
	*n* = 11	*n* = 14	*n* = 20	
Abdominal pain	0 (0)	3 (21)	12 (60)	0.0001 ^μ,‡^
Bloating	3 (27)	7 (50)	8 (40)	0.2
Pyrosis	3(27)	5 (36)	6 (30)	0.7
Nausea	0(0)	2 (14)	8 (40)	0.005 ^‡^
Vomiting	0(0)	2 (14)	3 (15)	0.2
Constipation	3(27)	7 (50)	14 (70)	0.03 ^‡^
Diarrhea	1(9)	5 (36)	8 (40)	0.1
Anorexia	3(27)	5 (36)	11 (55)	0.2

GIS, gastrointestinal symptoms. Comparison made during the second measurement with Xi^2^ and Fisher exact tests. ^µ^ *p* ≤ 0.01, Comparison Moderate vs. Severe ^‡^ *p* ≤ 0.05, Comparison Mild vs. Severe.

**Table 3:** Relationship of nutritional parameters and the severity of GIS.

	Intensity of Gastrointestinal Symptoms			
Parameters	Mild *n* = 11	Moderate *n* = 14	Severe *n* = 20	*p* **
**Nutritional Parameters**				
Weight (kg)	65.4 ± 12	73.2 ± 10.7	65.8 ± 16.7	0.4
BMI (kg/m^2^)	27 ± 4.6	27.8 ± 3.3	25.7 ± 4.4	0.4
^∞^ Triceps skinfold (mm) ^a^	14 (10–26)	11 (2–30)	10 (4–20)	0.06
Arm circumference (cm)	28 ± 5	28.3 ± 2.2	27.2 ± 4.3	0.8
Muscle arm circumference (cm)	23 ± 3.9	24.2 ± 2.2	23.7 ± 3.8	0.8
^∞^ Dynamometry (kg) ^b^	15.5 (10–38)	17 (12.5–33)	23.2 (12–40)	0.1
**Laboratory Parameters**				
BUN (mg/dL)	60 ± 15	72 ± 21	61.6 ± 26	0.3
Creatinine (mg/dL)	8 ± 4	10.9 ± 3.7	7.9 ± 2.4	0.05
Albumin (g/dL)	3.3 ± 0.8	3.5 ± 0.5	3.3 ± 0.4	0.6
Hemoglobin (g/dL)	11.3 ± 0.9	11.1 ± 1.8	10.7 ± 1.5	0.6
TIBC	241 ± 22	249 ± 68	266 ± 48	0.5
**Energy and Nutrient Intake**				
Energy (kcal/day)	1225.6 ± 372	1319 ± 264	1564 ± 638	0.1
Energy (kcal/kg)	20 ± 8.6	18.6 ± 5	25 ± 11	0.1
Protein (g/day)	61 ± 17	51.4 ± 14	65 ± 24	0.1
Protein (g/kg)	0.98 ± 0.2	0.65 ± 0.2	1.0 ± 0.4	0.03
Lipids (g/day)	36 ± 18	39 ± 18	42 ± 24	0.7
Carbohydrates (g/day)	164 ± 46	187 ± 44	220 ± 100	0.09
**Bioelectrical Impedance**				
R (Ω)	526 ± 122	445 ± 52	514 ± 99	0.09
Xc (Ω)	40 ± 10	34 ± 10.7	35.6 ± 11.5	0.5
R/H (Ω/m)	342 ± 86	275 ± 35	324.8 ± 7	0.07
Xc/H (Ω/m)	26 ± 7	20.9 ± 6.4	22.5 ± 7.6	0.3
Phase angle	4.4 ± 0.7	4.3 ± 1	4 ± 1	0.6

BUN, blood urea nitrogen; TIBC, total iron-binding capacity; R, resistance; Xc, reactance; R/H, resistance/height; Xc/H, reactance/height; Ω, Ohm. Data expressed as number (percentages), mean ± standard deviation and medians (interquartile ranges) when specified ^∞.^ ** One-way ANOVA and Kruskal Wallis for non-parametric variables. Comparisons made during the second measurement. ^a^ *p* < 0.05, Comparison Moderate vs. Severe. ^b^ *p* < 0.05, Comparison Mild vs. Severe.
